# Inhibitory effects of local anesthetics on the proteasome and their biological actions

**DOI:** 10.1038/s41598-017-04652-2

**Published:** 2017-07-11

**Authors:** Udin Bahrudin, Masaki Unno, Kazuya Nishio, Akiko Kita, Peili Li, Masaru Kato, Masashi Inoue, Shunichi Tsujitani, Takuto Murakami, Rina Sugiyama, Yasushi Saeki, Yuji Obara, Keiji Tanaka, Hiroshi Yamaguchi, Isao Sakane, Yasushi Kawata, Toshiyuki Itoh, Haruaki Ninomiya, Ichiro Hisatome, Yukio Morimoto

**Affiliations:** 1 Institute of Regenerative Medicine and Biofunction, Graduate School of Medical Science, Tottori University, Yonago, Japan; 2 Graduate School of Science and Engineering, Ibaraki University, Hitachi, Ibaraki Japan; 3 Frontier Research Center for Applied Atomic Sciences, Ibaraki University, Tokai, Ibaraki Japan; 4 Research Reactor Institute, Kyoto University, Kumatori, Osaka Japan; 5 Department of Molecular Medicine and Therapeutics, Faculty of Medicine, Tottori University, Yonago, Japan; 6 Department of Surgery, National Hospital Organization Kure Medical Center, Kure, Hiroshima Japan; 7 Cancer Center, Tottori University Hospital, Yonago, Japan; 8 School of Science and Technology, Kwansei Gakuin University, Sanda, Hyogo Japan; 9 RIKEN SPring-8 Center, RIKEN Harima Institute, Sayo, Hyogo Japan; 10 Laboratory of Protein Metabolism, Tokyo Metropolitan Institute of Medical Science, Tokyo, Japan; 11 Department of Chemistry and Biotechnology, Graduate School of Engineering, Tottori University, Tottori, Japan; 12 Department of Biological Regulation, Tottori University, Yonago, Japan; 13 Department of Cardiology and Vascular Medicine, Faculty of Medicine, Diponegoro University, Semarang, Central Java Indonesia; 14 Graduate School of Life Science, University of Hyogo, Kamigori, Hyogo Japan

## Abstract

Local anesthetics (LAs) inhibit endoplasmic reticulum-associated protein degradation, however the mechanisms remain elusive. Here, we show that the clinically used LAs pilsicainide and lidocaine bind directly to the 20S proteasome and inhibit its activity. Molecular dynamic calculation indicated that these LAs were bound to the β5 subunit of the 20S proteasome, and not to the other active subunits, β1 and β2. Consistently, pilsicainide inhibited only chymotrypsin-like activity, whereas it did not inhibit the caspase-like and trypsin-like activities. In addition, we confirmed that the aromatic ring of these LAs was critical for inhibiting the proteasome. These LAs stabilized p53 and suppressed proliferation of p53-positive but not of p53-negative cancer cells.

## Introduction

Local anesthetics (LAs) are clinically important for treatment of arrhythmias, control of pain, and suppression of seizures^1–3^. They work by blocking voltage-gated Na^+^ channels responsible for initiating action potentials in excitable cells to cause the electrical conduction block of the heart and neurons as their acute effect^4–7^. LAs reach the binding site within the channel pore between the selective filter and the channel gates to modify the channel conformation through interactions of their aromatic ring with the aromatic group of amino acid residues located in the Na^+^ channel gate^8, 9^.

Recently, LAs such as quinidine, mexiletine and aprindine have been reported to stabilize Na^+^ channels (Nav1.5)^10, 11^ as well as human ether-go-go-related gene (hERG) K^+^ channels^12^, and Kir6.2 proteins^13^, when administered for a long time. Channel proteins with a short half-life time such as Nav1.5^14^, cystic fibrosis transmembrane regulator^15^, gap junction channel connexin 43^16, 17^, Kv1.5^18^, Kir6.2^13^, and hERG^12^ are degraded through the ubiquitin-proteasome system (UPS). LAs have been suggested to have some effects on the UPS based on the results of microarray analyses showing up-regulation of channel proteins with a short half-life time by these agents^19^.

P53 is the key molecule for regulating cell proliferation, which is degraded through UPS^20^, and its accumulation is well-known to induce cellular apoptosis^21^. Among the anticancer properties of various medicines, it is reported that they modulate the expression of p53 and induce apoptosis, thus LAs-induced modification of the expression of p53 may contribute to developing the new anticancer agents. On the other hand, an increased expression of p53 may explain their harmful effects on the heart. An increase in p53 induces left ventricular dilatation and dysfunction in mice defficient in MDM2, an E3 ligase for p53. Furthermore, it has been demonstrated that p53 is critically involved in pressure overload–induced cardiac dysfunction^22^. Indeed, in clinical settings, such as in the Cardiac Arrhythmia Suppression Trial (CAST study)^23^, for preventing arrhythmias, long term treatment with Na^+^ channel blockers was reported deterioration of the cardiac function accompanied by death of patients with myocardial infarction, suggesting Na^+^ channel blockers may show deleterious effects on the heart via affecting proteasome activity. Thus, aiming at the development of new anticancer agents as well as at the attenuation of the harmful action of LAs, we explored the action of LAs on the proteasome.

LAs commonly have an aromatic ring with an amido bond. LAs can share this common structure with authentic proteasome inhibitors targeting the chymotrypsine-like site of the 20S proteasome such as MG132 and PS-341^24^, suggesting that the aromatic ring of the LAs may interact not only with Na^+^ channels but also with the chymotrypsine-like site of the 20S proteasome. Thus, LAs can access the chymotrypsine-like site of the 20S proteasome to inhibit it. The 20S proteasome consists of four heptameric rings (two outer α rings and two inner β rings), which are made up of seven structurally related α and β subunits, respectively, displaying an α_1–7_β_1–7_β_1–7_α_1–7_ organization^25, 26^. Three β1, β2, and β5 subunits of the inner β-rings contain catalytically active threonine residues at their N termini, in which the β1, β2, and β5 subunits are associated with caspase-like, trypsin-like, and chymotrypsin-like activities, respectively. Two pairs of these three active sites face the interior of the cylinder and reside in a chamber formed by the two β rings.

The initial purpose of the present study was to determine whether and how the LAs inhibit the UPS to stabilize the short-lived proteins degraded via proteasomes. We identified the structure-based mechanism of LAs binding to the chymotrypsine-like site of the β5 subunit of the 20S proteasome along with the common structure to exert its Na^+^ channel blocking action. We also demonstrated that LAs suppress the proliferation of MKN45 cells.

## Results

### Inhibition of the 20S Proteasome Activity

**Figure 1:**
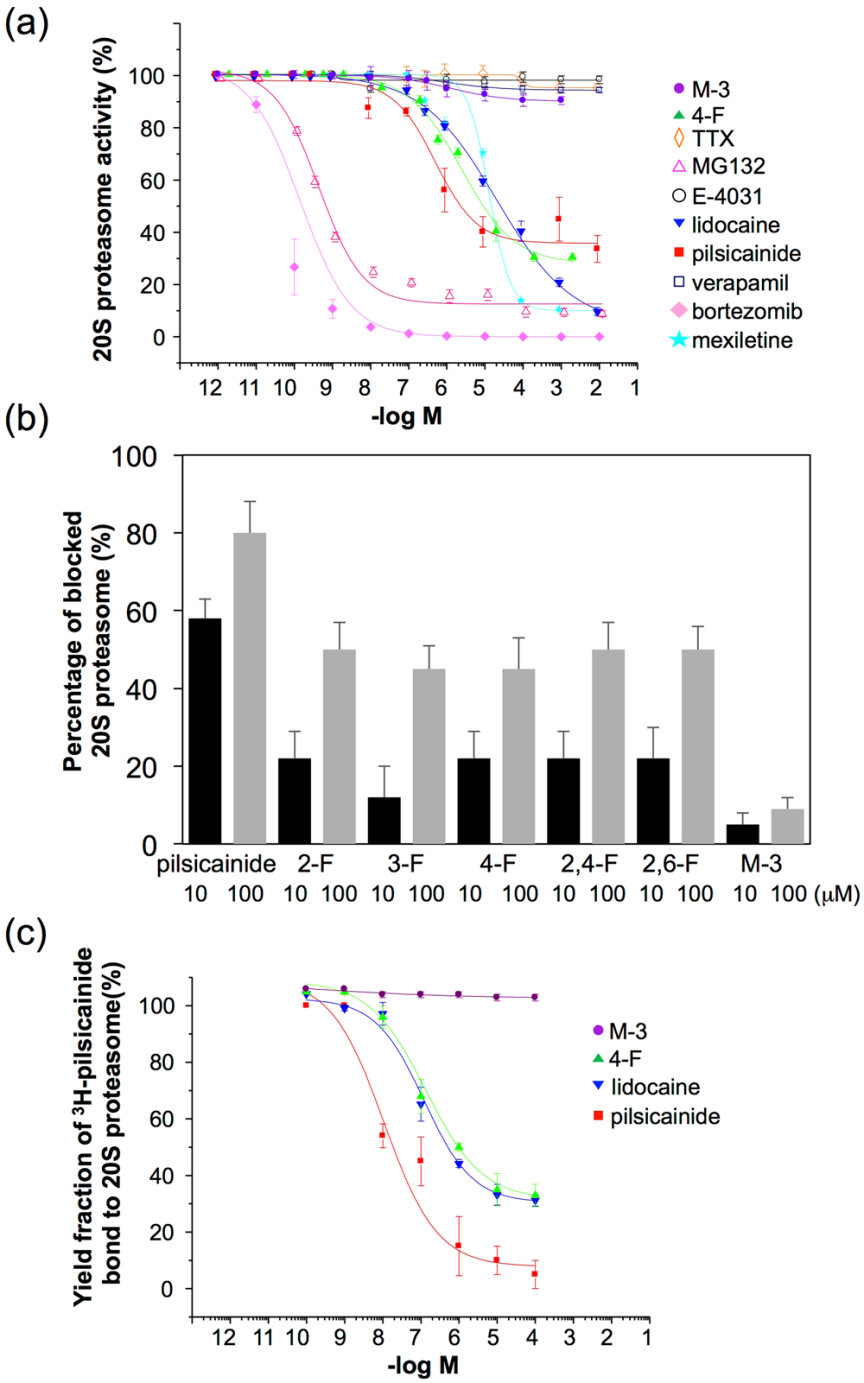
Effects of LAs on 20S proteasome activity and binding to the 20S proteasome. (**a**) Effects of LAs and other related chemical agents (■: pilsicainide; ▼: lidocaine; ∆: MG132; ♦: bortezomib; □: verapamil; ○: E-4031; ◊: TTX; ▲: 4-F; ●: M-3) on 20S proteasome activity *in vitro*. Each point represents the mean ± SEM of more than 7 determinations. 20S proteasome activities were expressed relative to their values in the absence of drugs (100%). The calculated IC_50_ values were 4.1 ± 2.4 nM, 1.1 ± 1.8 nM, 3.5 ± 1.5 μM, 23 ± 0.2 μM, 20 ± 2.5 μM, 14 ± 6.3 μM for MG132, bortezomib, pilsicainide, lidocaine, 4F, and mexiletine, respectively. (**b**) Inhibitory action of pilsicainide, 2-F, 3-F, 4-F, 2,4-F, 2,6-F and M-3 at 10 μM (left) and 100 μM (right) on the chymotrypsin-like activity of the proteasome. (**c**) Inhibition of binding of [^3^H]-pilsicainide to the 20S proteasome by cold pilsicainide, 4-F, M-3 and lidocaine *in vitro*. Each point represents the mean ± SEM of more than 7 determinations. The binding fraction of [^3^H]-pilsicainide to the 20S proteasome was expressed relative to the values in the absence of cold pilsicainide, 4-F, M-3 or lidocaine (100%). *K*_d_ values for pilsicainide, lidocaine, and 4-F are1.0 ± 2.8 μM, 21.2 ± 3.5 μM, and 4-F, respectively, whereas no binding capacity of M-3 was observed.

In an attempt to clarify the mechanism of action of LAs, we examined whether LAs inhibited the activity of the 20S proteasome *in vitro*. LAs with an aromatic ring, such as pilsicainide and lidocaine, inhibited 20S proteasome activity although the extent of inhibition was significantly lower than that attained with MG132 or bortezomib (Fig. 1a). IC_50_ values were 4.1 ± 2.4 nM, 1.1 ± 1.8 nM, 3.5 ± 1.5 μM, 23 ± 0.2 μM, 20 ± 2.5 μM, and 14 ± 6.3 μM for MG132, bortezomib, pilsicainide, lidocaine, 4-F, and mexiletine, respectively (Explanation of 4-F is described later and supplementary information). Neither the Ca^2+^ channel blocker verapamil which contains two aromatic rings, nor the K^+^ channel blocker E-4031, affected the activity of the 20S proteasome (Fig. 1a). Tetrodotoxin (TTX), a Na^+^ channel blocker without an aromatic ring, did not inhibit 20S proteasome activity, either (Fig. 1a and Supplementary Fig. S1), suggesting that the single aromatic ring of LAs might play a role in the blockade of proteasome activity. Actually, quinidine, mexiletine and aprindine have a single aromatic ring similar to lidocaine and pilsicainide, and both aprindine and mexiletine inhibit the 20S proteasome, although we previously reported that bepridil (Supplementary Fig. S1), a Na^+^ channel blocker with two aromatic rings, did not show the inhibitory action on the proteasome^27^. To substantiate this idea, we examined whether modification of the aromatic ring of LAs could also affect 20S proteasome activity. Fluorinated derivatives 2-F, 3-F, 4-F, 2,4-F and 2,6-F with altered electronic properties of the compound^28, 29^ (Supplementary Table S1 and Fig. S1) partially inhibited the chymotrypsin-like activity of the 20S proteasome (Fig. 1a,b), while M-3 did not (Fig. 1a,b). IC_50_ values for 2-F, 3-F, 4-F, 2,4-F and 2,6-F were 21.5 ± 46.8 μM, 20.5 ± 21.8 μM, 23 ± 0.2 μM, 23 ± 22 μM, and 28 ± 33 μM, respectively. We also conducted a [^3^H]-pilsicainide binding assay in the absence and presence of pilsicainide, lidocaine, 4-F as well as M-3. The [^3^H]-pilsicainide binding assay revealed binding of pilsicainide, lidocaine, and 4-F to the 20S proteasome with *K*_d_ = 1.0 ± 2.8 μM, *K*_d_ = 21.2 ± 3.5 μM and *K*_d_ = 20 ± 3.2 μM, respectively, whereas no binding capacity of M-3 was observed (Fig. 1c).

### Docking Simulations of LAs binding to the 20S proteasome

**Figure 2:**
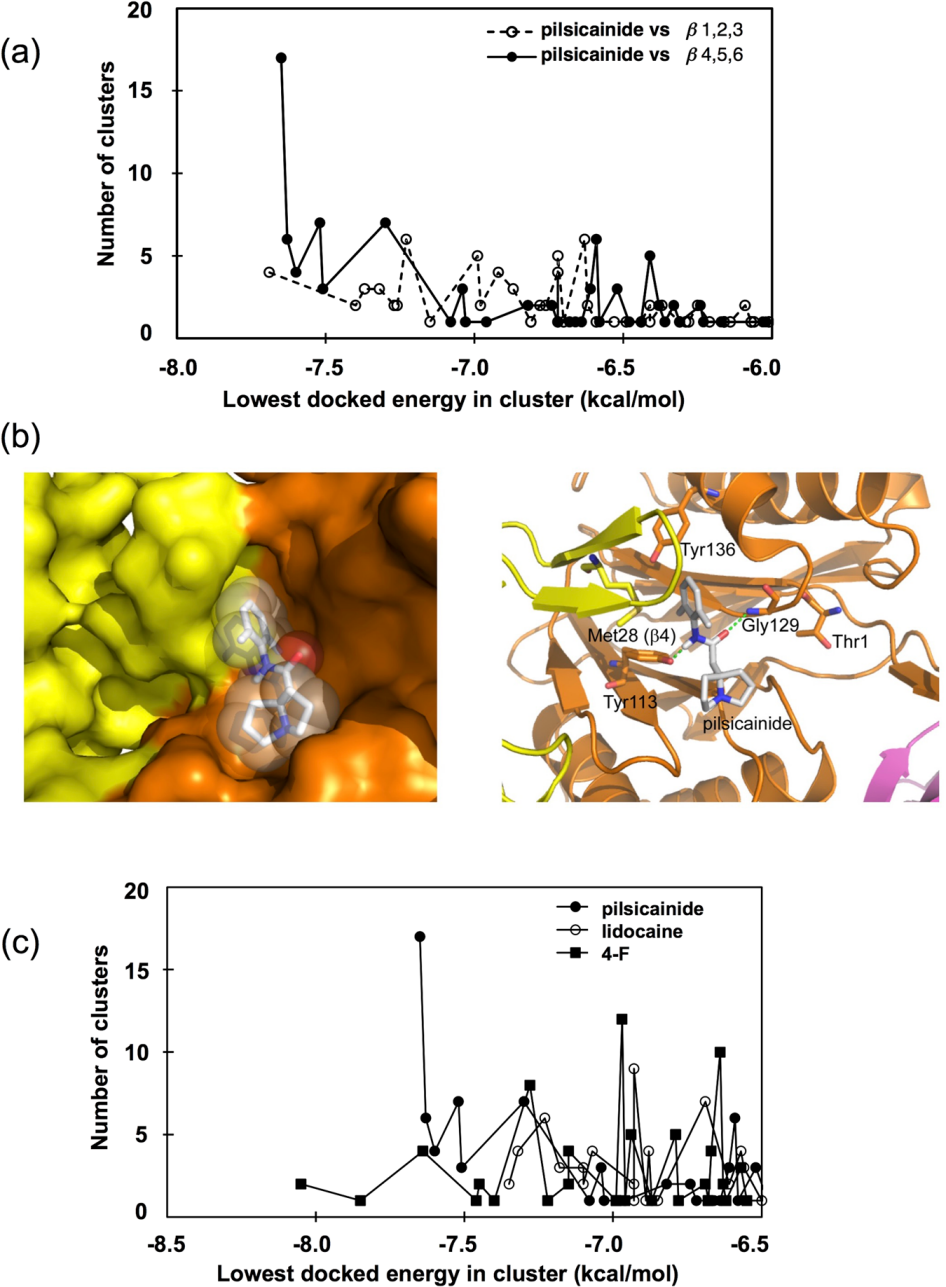
Predicted binding mode of LAs to the proteasome. (**a**) Docking simulations of pilsicainide against two parts of the β-subunit ring. (**b**) The binding mode of pilsicainide in the β5 subunit. The approximate position of S1 site pocket is designated. CPK model of pilsicainide and the surrounding protein matrix surface (left panel). Subunits β4 and β5 are colored yellow and orange, respectively. Amino acid residues surrounding the pilsicainide molecule and the nucleophilic active site residue of Thr1 are represented by the stick model and named (right panel). Green broken lines denote putative hydrogen bonds between the ligand and the protein. **(c)** Docking simulations of pilsicainide and its derivative 4-F, and lidocaine against β4-β5-β6 subunits.

Automated docking simulations were performed to find the binding mode of pilsicainide to the active site region of bovine proteasomes. Hetényi and van der Spoel have reported a considerable reliability automated docking simulations with the program AutoDock 3.0 in predicting the binding mode of a ligand molecule to a protein without prior knowledge about its binding site^30^. In this study, we have adopted a two-step method to search a plausible binding site of pilsicainide: firstly, binding results were classified into clusters roughly by their positions and secondly, a cluster was selected that was significantly populated and contained a binding conformation of low energy. Docking simulations of pilsicainide against two segments of the β ring (β1-β2-β3 section and β4-β5-β6 section) showed a significantly low docked energy in a conformational cluster in β4-β5-β6 but not in β1-β2-β3 (Fig. 2a). The energetically most stable and most populated cluster was found in the pocket-like structure of the β5 subunit (Fig. 2b). For pilsicainide binding, one sufficient cluster was found in a grid map around the β5 subunit and no sufficient clusters were found for a grid map around β4 and β6. The cluster found in a grid map around β5 contained 17-binding conformations, including a binding conformation with the lowest energy. The binding mode and the binding conformation with the lowest energy are shown in Fig. 2b. Through interactions of its aromatic ring with the aromatic residue of Tyr136 and of its amido group with both Tyr113 and Gly129, pilsicainide occupied a groove (S1′ pocket) formed by three β-strands at the subunit interface of β4 and β5; i.e., Val26-Met28 of β4, Arg120-Ser122 and Thr125-Val128 of β5, which is located in the vicinity of the nucleophilic attacking residue Thr1 of the subunit (S1 pocket). The *K*_I_ value (dissociation constant of inhibitor) estimated from the binding energy was determined to be 1 μM, which was a little lower than that from the binding experiment. In contrast to pilsicainide, which showed a well populated and low binding-energy cluster, other analogs tested did not showed such a significant binding clusters. The 4-F derivative was energetically favored but showed less populated clusters against the β4-β5-β6 section compared to pilsicainide, while lidocaine showed only less energetically favored and less populated clusters compared to pilsicainide (Fig. 2c). The conformation of the lowest binding energy in the cluster that lays in the binding pocket showed binding-free energy and a *K*_I_ value for lidocaine of −7 kcal/mol and 20 μM (Fig. 2a and c), respectively. This suggests that lidocaine has less affinity to the binding site of pilsicainide, which was consistent with the results from their inhibitory action on 20S proteasome activities.

Trypsin-like and caspase-like activities of the wild-type 20S proteasome were not inhibited by pilsicainide (Supplementary Fig. S2). These results were in accordance with the finding that pilsicainide binds only to the β5 catalytic subunit of the 20S proteasome.

### Stabilization of Proteins by LAs and MG132

**Figure 3:**
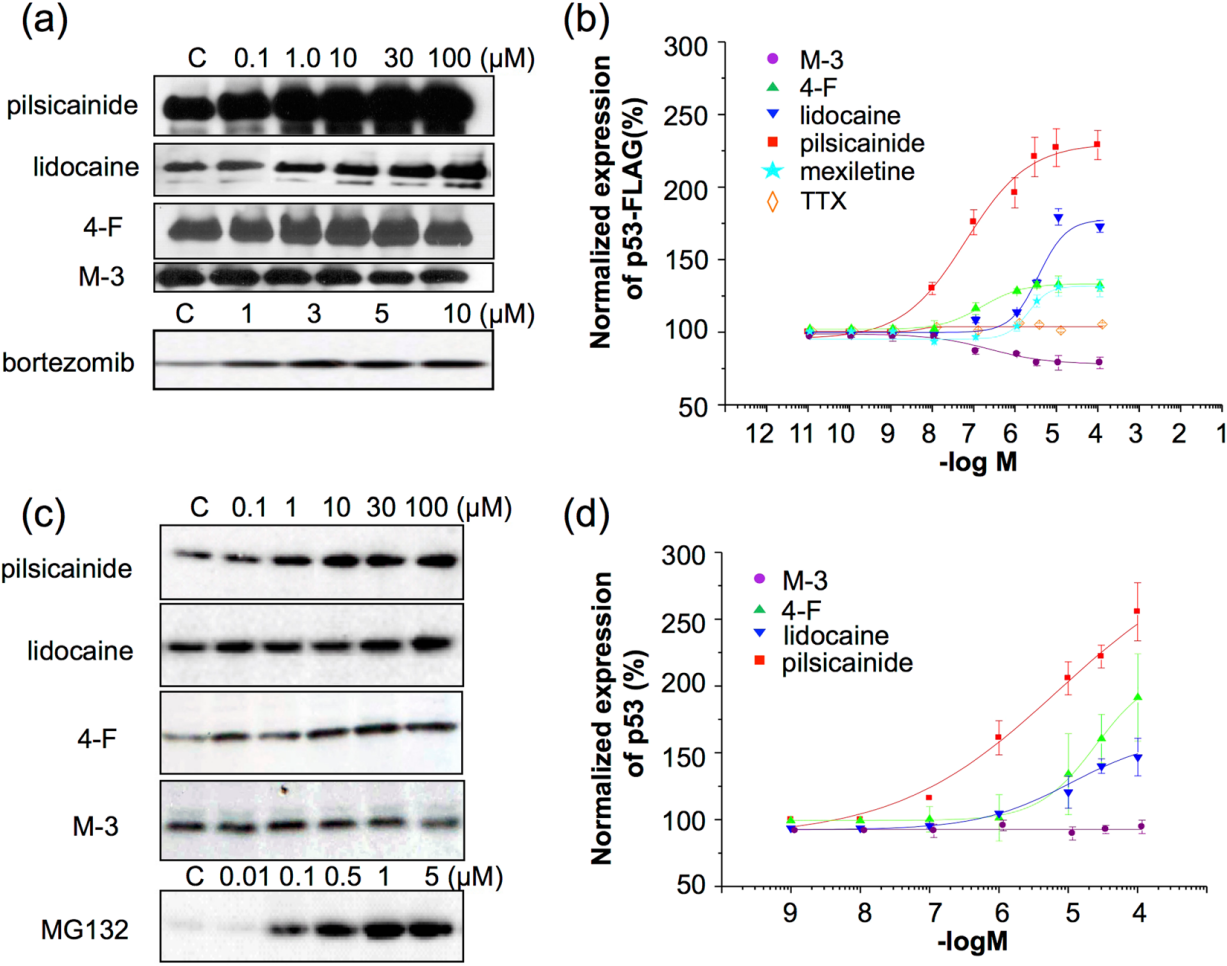
Pilsicainide and lidocaine increase transfected and endogenous p53. (**a**), (**b**) Dose-dependent effects of MG132 and LAs on ubiquitinated p53-FLAG protein. Cell lysates were analyzed by Western blot analysis (**a**). Quantitative analysis of p53-FLAG stability using densitometry is shown in (**b**). Each point was expressed relative to the values in the absence of drugs (100%). (**c**), (**d**) Dose-dependent effects of LAs on endogenous p53 expression in MKN45 cells. MKN45 cells were cultured for 24 h in the presence of pilsicainide, lidocaine, 4-F, M-3 or MG132. Cell lysates were analyzed by Western blot analysis (**c**). Quantitative analysis of the protein level of endogenous p53 was conducted using densitometry (**d**).

Inhibition of the 20S proteasome results in stabilization of proteins degraded through the UPS. The tumor suppressor p53 is well known to be degraded through the UPS. Like MG132, both pilsicainide and lidocaine increased the expression of p53-FLAG in COS7 cells together with a concomitant accumulation of its ubiquitinated form (Supplementary Fig. S3a). This p53 is ubiquitinated primarily by E3 ligase Mdm2^31^ and its C438A mutant works as a dominant negative form. Co-expression of the C438A mutant of Mdm2 with p53-FLAG abolished the effects of pilsicainide, lidocaine and MG132, providing further evidence for proteasome inhibition by these LAs (Supplementary Fig. S3b). 4-F partially increased the protein expression of p53-FLAG, while M-3 failed to affect it (Fig. 3a,b).

Given these results, we examined the effects of LAs on the proliferation of MKN45 gastric cancer cells that express p53. Both pilsicainide and lidocaine increased the p53 protein level in MKN45 cells in a dose-dependent manner. 4-F increased p53 a little and M-3 failed to increase it (Fig. 3c,d).

### Suppression of Proliferation of Cancer Cells

**Figure 4:**
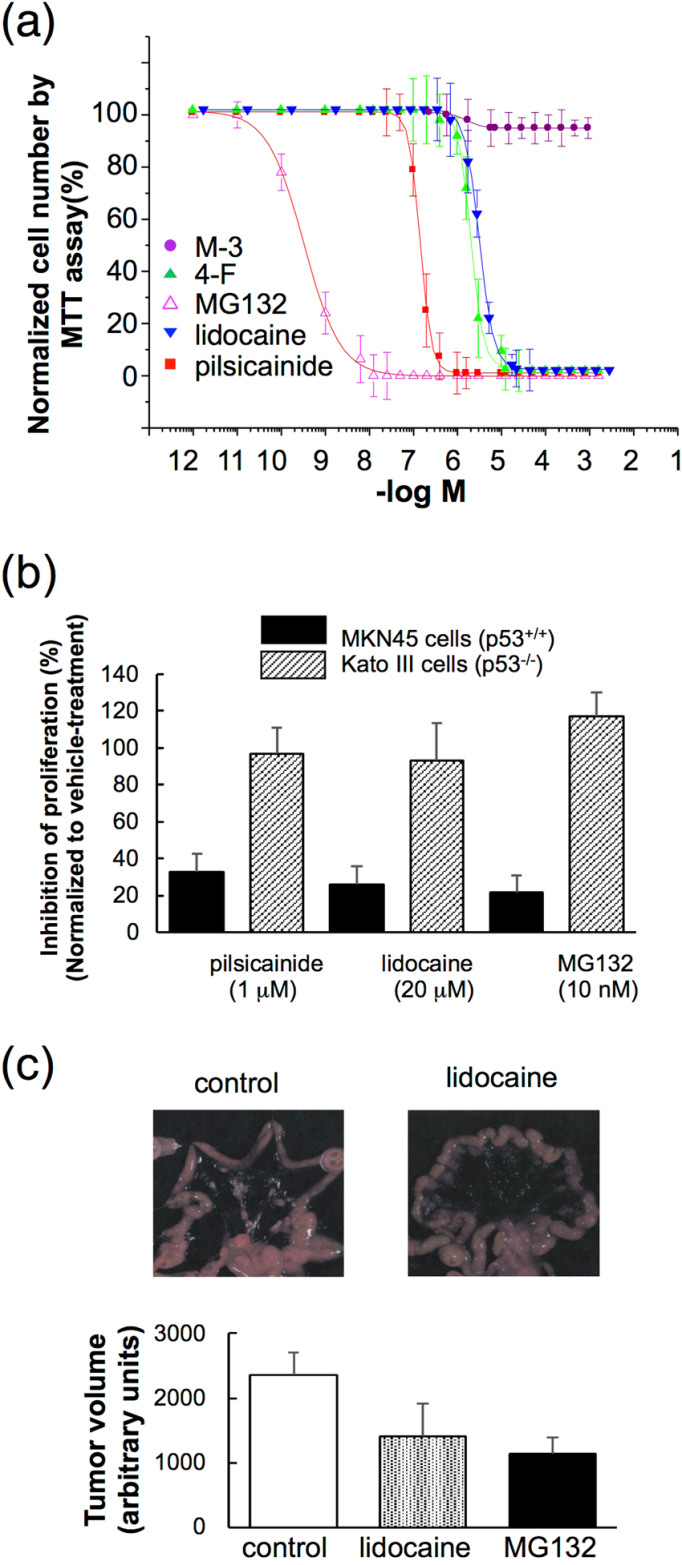
Effects of LAs on cancer cells. (**a**) Effects of LAs on the proliferation of MKN45 cells. MKN45 cells were cultured for 24 h in the presence of MG132, pilsicainide, lidocaine, 4-F or M-3. Proliferation of MKN43 was estimated by MTT assays. (**b**) Effects of LAs on the proliferation of MKN45 and Kato III cells cultured for 24 h in the presence of MG132 (10 nM), pilsicainide (1 μM) and lidocaine (20 μM). (**c**) In the *in vivo* experiments, lidocaine significantly decreased the proliferation of MKN45 cells dispersed among mouse mesenchymal cells to a similar extent as MG132.

Furthermore, pilsicainide and lidocaine suppressed proliferation of MKN45 cells in a dose-dependent manner, whereas M-3 did not inhibit their proliferation (Fig. 4a). These effects of pilsicainide and lidocaine were not observed in Kato III gastric cancer cells that do not express p53 (Fig. 4b). To test the inhibitory action of LAs on the proliferation of MKN45 cells *in vivo*, MKN45 cells were pretreated with lidocaine and then transplanted into the intestine of SOD SKID mice. Lidocaine treatment resulted in suppression of tumor growth (Fig. 4c).

## Discussion

In this work, we demonstrated that LAs stabilized the short-lived protein degraded in the cytosol by proteasomes, and LAs with an aromatic ring, such as pilsicainide, bound directly to the proteasome β5 subunit and inhibited its chymotrypsin-like activity, although the affinity to S1′ pocket differed between pilsicainide and lidocaine. Modification of the aromatic ring of pilsicainide attenuated its inhibitory action on the 20S proteasome.

The eukaryotic 20S proteasome has at least three different types of the specificity against substrate polypeptides, i.e., caspase-like (for acidic amino acid residues), trypsin-like (for basic amino acid residues), and chymotrypsin-like (for hydrophobic and aromatic amino acid residues) specificities^32^. These three specificities have been assigned to three active subunits β1, β2, and β5, respectively^33^, determined by using synthesized fluorogenic peptides *in vitro*. The subunit β5 has the chymotrypsin-like activity and mutations in the active site in this subunit were shown to be lethal or to disturb cell growth^33^. The predicted binding site of pilsicainide was apart from the nucleophilic attacking residue Thr1 of the subunit. From crystallographic^25, 34^ and modeling studies^35^, it was reported that inhibitors specific for the proteasome mainly targeted the S1 site.

In our estimated model, however, pilsicainide bound to the groove that is located in the vicinity of S1 site, as shown in Fig. 2. This groove is presumably acting as the S1′ site of proteinase as an analogy of chymotrypsin, and the inhibitor is disturbing the substrate binding. Thus, this S1′ site could be another target for designing a new inhibitor for the proteasome. The inhibitory action of LAs on the chymotrypsin-like activity of the 20S proteasome may come from their common structure. LAs essentially bear an aromatic ring and an amido group in their structure not only for conferring lipophilicity to a drug that allows it to pass through membranes, but also for CH-π or π-stacked binding to the aromatic group of amino acid residues to block Na^+^ channels activities. Our estimated model demonstrated that pilsicainide bound to the S1′ site via interaction of its aromatic ring with Tyr136 (CH-π or π-stacked binding) and/or interaction with the amido group of Tyr113 and Gly129 (hydrogen bonding). It is well-known that fluoride closely mimics the steric requirement of hydrogen at the enzyme receptor site, but its strong electronegativity significantly alters the reactivity of neighboring centers. Therefore, fluorinated derivatives have been a tool for the investigation of molecular interactions of the chemicals with amino acid residues. Since aromatic residues are capable of forming π-electron interactions with the aromatic drug portion, which are known to be crucial for high-affinity LA block of Na^+^ channel, aromaticity is crucial for Na^+^ channel function and pharmacology. Fluorinated derivatives of pilsicainide revealed a decrease in electron-density of their aromatic ring, which perturb not only the interaction with the aromatic group of Tyr136, but also interaction between the amido groups of Tyr113 and Gly129 (Fig. 2b), leading to less potent inhibition of the 20S proteasome activity (Fig. 1b) in comparison with pilsicainide. Thus, either the M-3 compound analogue of pilsicainide or their fluorinated derivatives attenuated both their bind to and block of the 20S proteasome (Fig. 1c), as well as their stabilization of p53-FLAG (Fig. 3). Taken together, the aromatic ring of LAs played a pivotal role by occupying the groove that is located in the vicinity of S1 site to interfere with the access of hydrophobic and aromatic amino acid residues of the targeted peptide to this site.

In this study, we found that LAs with an aromatic ring such as pilsicainide and lidocaine inhibit the proteasome activity. The 20S proteasome is a potential target in cancer therapy^36, 37^. However, these LAs are not very good inhibitors because their binding affinities were relatively low. Although based on the structure of pilsicainide, it will be possible to design anti-cancer drugs, that are hardly realized. Na^+^ channel blockers are used widely in the world for treating arrhythmias in patients with heart disease. According to the CAST study^23^, it is well-recognized that Na^+^ channel blockers significantly increase the mortality rate in patients with myocardial infarction, while its underlying mechanism remains unknown. Accumulation of p53 in the myocardium has been reported to be associated with chronic heart failure^38, 39^. Taken together, the present results that Na^+^ channel blockers, at clinical concentrations, increase p53 via inhibition of the proteasome, Na^+^ channel blockers could increase the mortality rate of patients with heart failure, which may explain in part the side effects observed in the CAST study. Therefore, the present study may contribute to the development of new selective Na^+^ channel blockers without inhibitory actions on the proteasome via modification of the aromatic ring, and safer for patients with heart failure. The clinical implications of this novel action of pilsicainide should be a subject of future investigations.

## Methods

### Plasmids and Expression

pcDNA3/p53-FLAG was a kind gift from Dr. Nakayama (Kyushu University). pcDNA3/Mdm2 (C438A) was a kind gift from Dr. Yasuda (Tokyo Pharmaceutical College). pCR/CMV/hH1 was a kind gift from Dr. Makita (Hokkaido University). COS7 cells were maintained in Dulbecco’s modified Eagle medium (Gibco BRL)/10% fetal bovine serum at 37 °C in a 5% CO_2_ incubator. Cells were transfected using lipofectamine (Gibco BRL) according to the instructions provided by the manufacturer. Forty-eight hours after transfection, cells were assayed. Proteasome inhibitors or LAs were applied 36 hours after transfection. All drugs except for MG132 were dissolved in culture medium or distilled water. MG132 was dissolved in dimethyl sulfoxide (DMSO). The final concentration of DMSO in the culture or reaction medium was equal to or less than 0.01% v/v. Pilsicainide was kindly provided by Daiichi Suntory Pharmaceutical Co., Ltd. (Osaka, Japan).

### Western Blot Analysis and Immunoprecipitation

The same amounts (10 μg) of protein were separated on SDS-PAGE and electro-transferred to a polyvinylidene difluoride membrane. Membranes were probed with antibodies against FLAG (1:1000, Cosmo Bio), GFP (1:1000, Molecular Probes), ubiquitin (1:1000, MBL) or β-actin (1:1000, Oncogene) and were developed using an ECL detection system. Immunoprecipitation was carried out in PBS/1% Triton X-100, 0.5% SDS, 0.25% sodium deoxycholate, 1 mM ethylenediaminetetraacetic acid (EDTA), and 10 μg/ml aprotinin, 10 μg/ml leupeptine, 10 μg/ml pepstatin and 1 mM phenylmethylsulfonylfluoride for 2 h at 4 °C. Immune complexes were collected with protein G agarose (GE Healthcare Life Science) and bound proteins were analyzed by SDS-PAGE followed by immunoblotting.

### Measurement of 20S Proteasome Activity

20S Proteasome activity was assessed by fluorescence of free AMC (7-Amino-4-methylcoumarin, excitation: 380 nm, emission: 460 nm) liberated from a substrate peptide (Suc-Leu-Leu-Val-Tyr-MCA). The reaction mixture contained the 20S proteasome (500 μg/ml), the substrate peptide (10 μM) and the indicated drugs in 25 mM HEPES, 0.5 mM EDTA, 0.03% SDS, pH 7.6. The mixture was incubated at 37 °C for 1 h. AMC fluorescence liberated in the absence of drugs was taken as the basal value (100%). The fluorescence liberated in the absence of SDS was taken as the background value (0%)^13^. Most of the activity assays were conducted using an assay kit (Calbiochem), but some were conducted in hand-made buffer solutions.

### Docking Simulation of Compounds of the Bovine 20S Proteasome

Docking simulations were performed using the AutoDock program package, version 3.0.5^40^. The crystal structure coordinates of the bovine proteasome were obtained from Brookhaven Protein Databank [PDB ID: 1IRU^26^]. The structure coordinates of the fourteen β subunits located in the inner rings of the proteasome were extracted from the data file for the docking simulation. Missing residues in the protein structure were reconstructed using the program Swiss-PDB Viewer^41^. Polar hydrogen atoms were reconstructed using the program ‘protonate’ supplied with AutoDock distribution. Atomic charges and atomic solvation parameters in the protein were assigned using the programs ‘q.kollua’ and ‘addsol’, respectively. The structure and Mulliken atomic charges of pilsicainide were calculated using Gaussian98 (Gaussian Inc., USA). In the ligand structure used for docking, freedom of torsion at the peptide bond was frozen and all non-polar hydrogen atoms were merged to their bonded carbon atom. Two grid maps were generated to cover three proteinase active subunits; β1, β2, and β5 (designated as H, I, and L in the PDB file, respectively). Grid maps were generated with 0.375 Å spacing within a rectangular volume of 38.6 Å × 47.6 Å × 47.6 Å. Default values were used for parameters of the simulation, except for the ‘maximum number of energy evaluations’ and ‘maximum number of generations’, both of which were set to 10^7^. The Lamarckian genetic algorithm with the pseudo Solis and Wets search was used for the calculations. The results of 100 individual trials were divided into clusters of conformations with an rms tolerance of 1.5 Å. These clusters were aligned with the docked energy of each cluster, which was determined as the lowest docked energy within the cluster. The cluster with a significant population and the lowest docking energy was selected as the set containing the most probable binding conformations. The conformation with the lowest docking energy within this set was selected as the most probable binding conformation.

### Ligand Binding Assay

Commercially available 20S proteasome was purchased from Calbiochem. LAs and their derivatives at increasing concentrations (10^–8^ to 10^–2^ M) were incubated with [^3^H]-pilsicainide for 1 h at room temperature in buffer containing 50 mM HEPES supplemented with 1% bovine serum albumin (pH 7.4). Following the reaction termination by addition of 100% tricarboxylic acid, the bound and unbound ligands were separated by centrifugation. Specific binding was defined as the difference between binding capacities in the pellet and supernatant.

### Analysis of Data on Dose-Dependent Inhibition of the 20S proteasome

Concentration dependence of the effects of each drug on the stability of the short-lived protein and the 20S proteasome activity was fitted using the following equation:

$${\rm{B}} \% ={{\rm{B}}}_{{\rm{\max }}}\cdot {[{\rm{Drug}}]}^{{\rm{n}}}/({{{\rm{IC}}}_{{\rm{50}}}}^{{\rm{n}}}+{[{\rm{Drug}}]}^{{\rm{n}}})$$

where, B% represents the changes in expression of the short-lived protein and the 20S proteasome activity induced by drugs at a given concentration of the [drug], and B_max_ represents the maximum attainable reduction. IC_50_ and n are the half-maximal inhibitory concentration and Hill coefficient, respectively.

One-way ANOVA test and Student-t test were carried out to compare the groups. All data are expressed as the mean ±SEM. The level of statistical significance was set at p < 0.05.

## Electronic supplementary material

Inhibitory effects of local anesthetics on the proteasome and their biological actions
