# Supplementary material for: Inhibitory effects of local anesthetics on the proteasome and their biological actions
Source: Sci Rep. 2017 Jul 11;7:5079. doi: 10.1038/s41598-017-04652-2 (PMC5506043; doi:10.1038/s41598-017-04652-2)
Supplement: Supplementary file 1 — Inhibitory effects of local anesthetics on the proteasome and their biological actions [file 41598_2017_4652_MOESM1_ESM.pdf]

Supplemental Information

Inhibitory effects of local anesthetics on the proteasome and their biological actions

Udin Bahrudin[1, †], Masaki Unno[2,3], Kazuya Nishio[4, ‡], Akiko Kita[4], Peili Li[1], Masaru Kato[5], Masashi Inoue[6], Shunichi Tsujitani[7], Takuto Murakami[8,9], Rina Sugiyama[2], Yasushi Saeki[10], Yuji Obara[2,3], Keiji Tanaka[10], Hiroshi Yamaguchi[8,9], Isao Sakane[11], Yasushi Kawata[11], Toshiyuki Itoh[11], Haruaki Ninomiya[12], Ichiro Hisatome[1, *], Yukio Morimoto[4, *]

[1]Institute of Regenerative Medicine and Biofunction, Graduate School of Medical Science, Tottori University

[2]Graduate School of Science and Engineering, Ibaraki University

[3]Frontier Research Center for Applied Atomic Sciences, Ibaraki University

[4] Research Reactor Institute, Kyoto University

[5]Department of Cardiology, Tottori University Hospital

[6]Department of Gastroenterological Surgery, National Hospital Organization Kure Medical Center

[7]Cancer Center, Tottori University Hospital

[8]School of Science and Technology, Kwansei Gakuin University

[9]RIKEN SPring-8 Center, RIKEN Harima Institute

[10]Laboratory of Protein Metabolism, Tokyo Metropolitan Institute of Medical Science

[11]Department of Chemistry and Biotechnology, Graduate School of Engineering, Tottori University

[12]Department of Biological Regulation, Tottori University

*Address correspondence to:
Ichiro Hisatome
Nishimachi 36-1, Yonago, Tottori 683-8503, Japan, hisatome@grape.med.tottori-u.ac.jp, FAX: +81-859-34-8099, Phone: +81-859-34-8101, or
Yukio Morimoto
Asashiro-Nishi 2, Kumatori, Osaka 590-0494, Japan, morimoto@rri.kyoto-u.ac.jp, FAX: +81-72-451-2371, Phone: +81-72-451-2371

[†]Present address: Department of Cardiology and Vascular Medicine, Faculty of Medicine, Diponegoro University Semarang, Central Java, Indonesia

[‡]Present address: Graduate School of Life Science, University of Hyogo

Synthesis of fluorophen y-methyl-free-pilsicainide

Reagents and solvents were purchased from common commercial sources and were used as received or purified by distillation over appropriate drying agents. Reactions requiring anhydrous conditions were carried out under argon with dry, freshly distilled solvents and magnetic stirring. Thin layer chromatography was performed with the indicated solvents and Wako gel B-5F. ^{1}H-NMR spectra and ^{13}C-NMR spectra were recorded on a JEOL JNM MH-270 or JNM MH-500 MHz spectrometer. Chemical shifts are expressed in ppm downfield from tetramethylsilane (TMS) with $CDCl_3$ as an internal reference. IR spectra were obtained on SHIMADZU FT-IR 8000 spectrometers. Point fluorinated analogues of pilsicainide, 4-F, 2-F, 3-F, and 2,6-F, were prepared following equation **(1)** and results of the synthesis are summarized in Table S1.

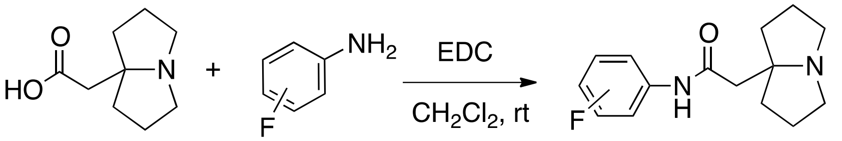

(1)

N-(4-fluorophenyl)-2-(hexahydro-1H-pyrrolizin-7a-yl)acetamide (4-F): To a dichloromethane (CH_2Cl_2) (2.0 mL) solution of 2-(hexahydro-1*H*-pyrrolizin-7a-yl)acetic acid (**1**) [30] (20.0 mg, 0.12 mmol), 1-(3-dimethylaminopropyl)-3-ethylcarbodiimide hydrochloride (EDC) (34.5 mg, 0.18 mmol) and 1-amino-4-fluorobenzen (26.7 mg, 0.24 mmol) were added at 0 °C, and the mixture was stirred for 24 h at 60 °C. After being cooled to room temperature (rt), the mixture was extracted with ethyl acetate and the combined organic layer was dried over anhydrous Na_2SO_4 and evaporated to dryness. Silica gel thin layer chromatography (TLC) yielded 76% acetamide 4-F (23.9 mg, 0.090 mmol); mp 170 - 180 °C (recrystallized from CH_2Cl_2);Rf 0.5 (CH_2Cl_2/ methanol = 3:1); ^{1}H NMR (270 MHz, CD_3OD, J = Hz) δ 2.03-2.30 (8H, m), 3.04 (2H, s), 3.19-3.33 (2H, m), 3.78 (2H, m), 7.00-7.11 (2H, m), 7.56-7.67 (2H, m); ^{13}C NMR (125 MHz, CD_3OD, J= Hz) δ 21.30, 34.14, 38.90, 53.60, 60.77, 77.56, 112.54 (d, $J_{C\text{-}F}$ = 22.1 Hz), 119.56 (d, $J_{C\text{-}F}$ = 8.2 Hz), 131.60, 157.03 (d, $J_{C\text{-}F}$ = 241.3Hz), 166.33; ^{19}F NMR (471 MHz, CD_3OD, J = Hz), δ 45.77; IR (KBr, cm^{-1}) 3269, 3071, 1665, 1618, 1506, 1404, 1234, 1209, 1103, 835, 785, 750; Anal. Calcd for $C_{15}H_{19}FN_2O$: C, 68.68; H, 7.30; N, 10.68. Found: C, 68.47; H, 7.04; N, 10.51.

Fluorine substituted acetamide 2-F, 3-F, 2,4-F, and 2,6-F were prepared following the same method.

N-(2-fluorophenyl)-2-(hexahydro-1H-pyrrolizin-7a-yl)acetamide (2-F): mp 240 - 260 °C (recrystallized from CH_2Cl_2); Rf 0.5 (CH_2Cl_2/ methanol = 3:1); ^{1}H NMR (270 MHz, D_2O, J = Hz) δ 1.87-2.07 (8H, m), 2.89 (2H, s), 3.00-3.11 (2H, m), 3.44-3.59 (2H, m), 7.04-7.22 (3H, m), 7.33-7.44 (1H, m); ^{13}C NMR (125 MHz, D_2O, J = Hz) δ 23.72, 36.48, 41.26, 56.16, 79.92, 116.11, 116.28, 124.82, 126.47, 128.51 (d, $J_{C\text{-}F}$ = 7.7 Hz), 155.73 (d, $J_{C\text{-}F}$ = 241.8Hz), 170.99; ^{19}F NMR (471 MHz, D_2O, J = Hz), δ 37.9; IR (KBr, cm^{-1}) 3447, 3298, 1686, 1616, 1506, 1458, 1259, 1192, 1103, 779; Anal. Calcd for $C_{15}H_{19}FN_2O$: C, 68.68; H, 7.30; N, 10.68. Found: C, 67.79; H, 7.32; N, 10.45.

N-(3-fluorophenyl)-2-(hexahydro-1H-pyrrolizin-7a-yl) acetamide (3-F): mp 290 - 300 °C (recrystallized from CH_2Cl_2); Rf 0.6 (CH_2Cl_2/ metanol = 3:1); ^{1}H NMR (270 MHz, CD_3OD, J =

Hz) δ 2.00-2.26 (8H, m), 3.00 (2H, s), 3.15-3.28 (2H, m), 3.70-3.85 (2H, m), 6.80-6.89 (1H, m), 7.22-7.37 (2H, m), 7.56-7.65 (1H, m); ^{13}C NMR (125 MHz, D_2O, J = Hz) δ 23.63, 36.39, 41.54, 54.04, 79.94, 109.04 (d, J_{C-F} = 25.9 Hz), 112.48 (m), 117.43, 130.71 (d, J_{C-F} = 10.1Hz), 137.97 (d, J_{C-F} = 12.0 Hz), 162.66 (d, J_{C-F} = 244.2 Hz), 170.38; ^{19}F NMR (471 MHz, D_2O, J = Hz), δ 49.50; IR (KBr, cm^{-1}) 3524, 3310, 1670, 1607, 1495, 1408, 1261, 1200, 1107, 858, 781, 667; Anal. Calcd for $C_{15}H_{19}FN_2O$: C, 68.68; H, 7.30; N, 10.68. Found: C, 67.51; H, 7.41; N, 10.41.

N-(2,4-difluorophenyl)-2-(hexahydro-1H-pyrrolizin-7a-yl) acetamide (2,4-F): Y = 89%; mp >300 °C (decomposed) (recrystallized from CH_2Cl_2); Rf 0.6 (CH_2Cl_2/ metanol = 3:1); ^{1}H NMR (270 MHz, CD_3OD, J = Hz) δ 2.04-2.30 (8H, m), 3.09 (2H, s), 3.19-3.33 (2H, m), 3.70-3.85 (2H, m), 6.90-7.10 (2H, m), 7.76-7.90 (1H, m); ^{13}C NMR (125 MHz, CD_3OD, J = Hz) δ 23.46, 36.32, 41.02, 55.86, 79.85, 104.63 (m), 111.71 (d, J_{C-F} = 25.92 Hz), 119.74 (d, J_{C-F} = 11.5 Hz), 127.87 (d, J_{C-F} = 10.6 Hz), 157.13 (d, J_{C-F} = 11.5 Hz), 166.33 (d, J_{C-F} = 11.5 Hz), 171.35; ^{19}F NMR (471 MHz, CD_3OD, J = Hz), δ 23.58, 23.63; IR (KBr, cm^{-1}) 3466, 2978, 1622, 1514, 1431, 1261, 1144, 1099, 966, 851, 808, 667; Anal. Calcd for $C_{15}H_{18}F_2N_2O$: C, 64.27; H, 6.47; N, 9.99. Found: C, 63.76; H, 6.38; N, 9.86.

N-(2,6-difluorophenyl)-2-(hexahydro-1H-pyrrolizin-7a-yl) acetamide (2,6-F): Y = 67%; mp 290 - 300 °C (recrystallized from CH_2Cl_2); Rf 0.5 (CH_2Cl_2/ metanol = 3:1); ^{1}H NMR (270 MHz, D_2O, J = Hz) δ 1.89-2.07 (8H, m), 2.94 (2H, s), 3.00-3.11 (2H, m), 3.48-3.59 (2H, m), 6.91-7.02 (2H, m), 7.19-7.33 (1H, m); ^{13}C NMR (125 MHz, D_2O, J = Hz) δ 23.46, 36.32, 40.77, 56.11, 79.85, 112.02 (d, J_{C-F} = 2.88 Hz), 112.18 (d, J_{C-F} = 2.88 Hz), 129.60 (d, J_{C-F} = 8.6 Hz), 157.00, 171.59; ^{19}F NMR (471 MHz, D_2O, J = Hz), δ 20.01, 20.22; IR (KBr, cm^{-1}) 3445, 3265, 1693, 1526, 1470, 1242, 1020, 806, 667; Anal. Calcd for $C_{15}H_{18}F_2N_2O$: C, 64.27; H, 6.47; N, 9.99. Found: C, 62.96; H, 6.56; N, 10.01.

Table S1. Synthesis of fluorinated-pilsicainide derivatives

Entry	Fluorinated-pilsicainide derivatives	Reaction Time (hour)	Yield (%)
1	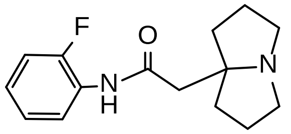 2-F	12	56
2	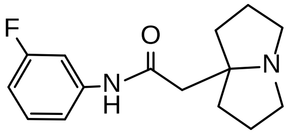 3-F	24	45
3	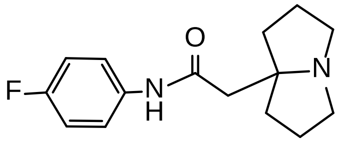 4-F	24	76
4	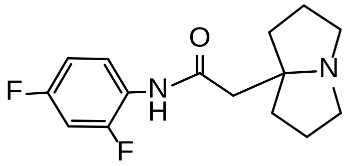 2,4-F	12	89
5	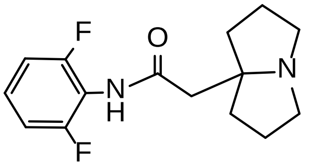 2,6-F	12	67

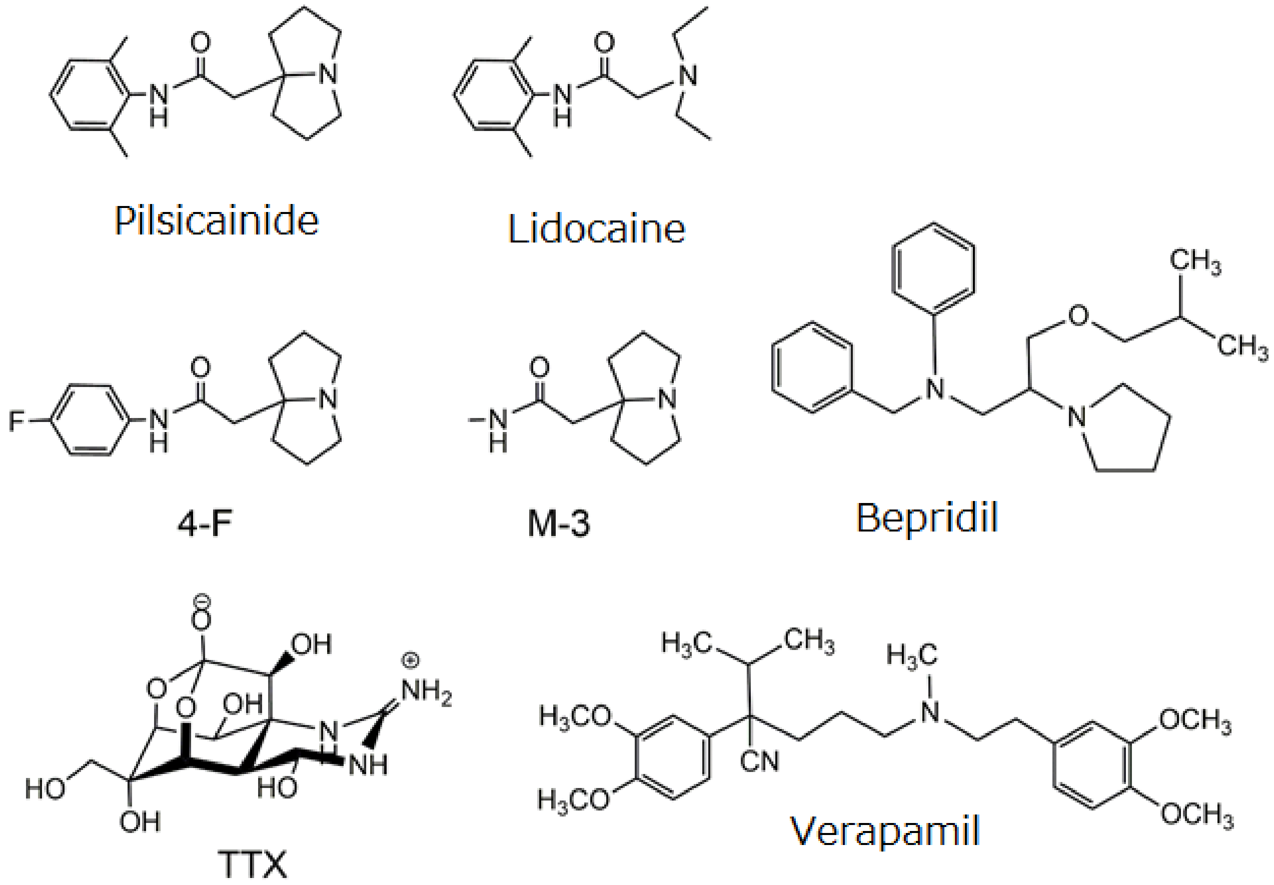

Fig. S1. Chemical structures of pilsicainide, lidocaine, 4-F, M-3, TTX, Bepridil, and Verapamil

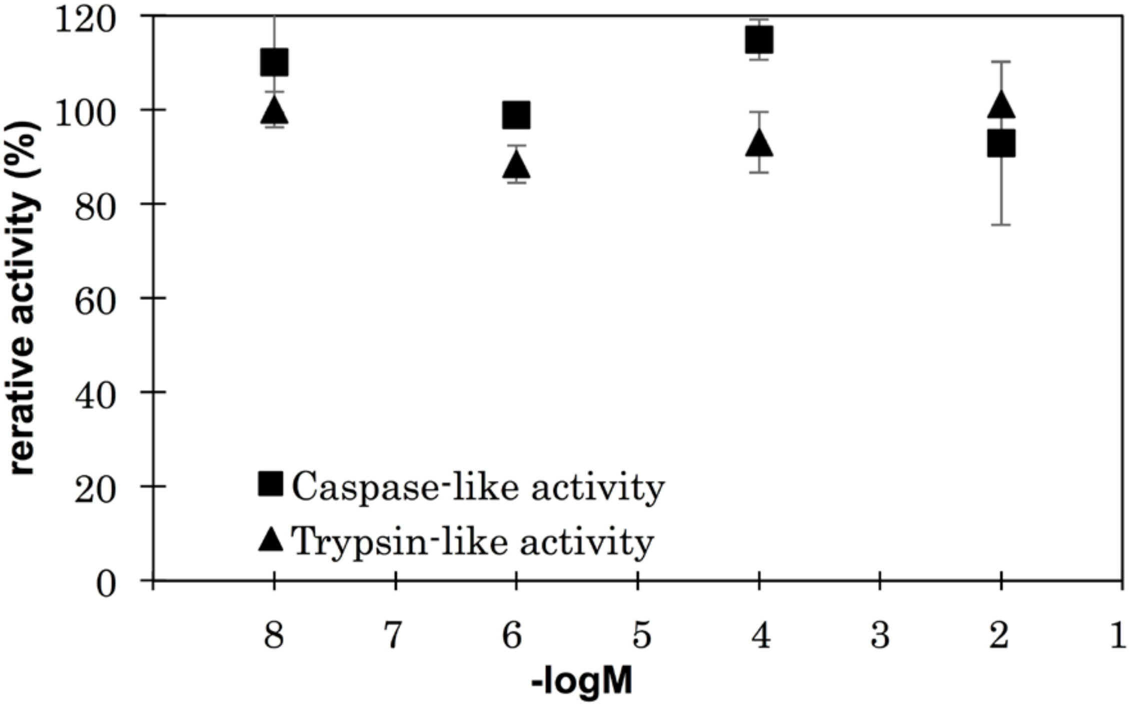

Fig. S2. Effects of pilsicainide on caspase- and trypsin-like activities of the 20S proteasome. The 20S Proteasome activities were expressed relative to their values in the absence of pilsicainide (100%). Each point represents the mean ± SEM of 3 determinations. ■: caspase-like activity, ▲: trypsin-like activity.

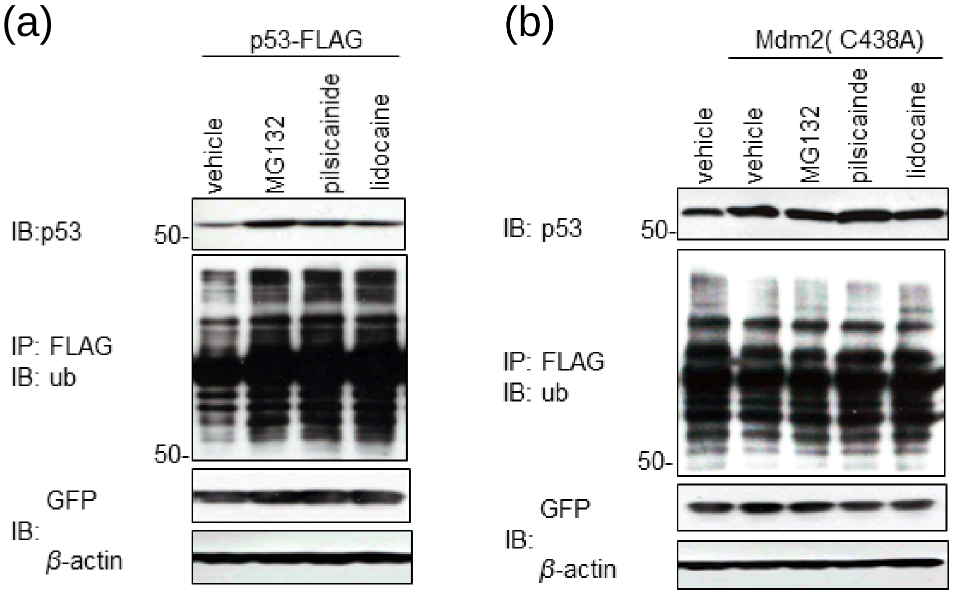

Fig. S3. Pilsicainide and lidocaine stabilize p53 through inhibition of the ubiquitin-proteasome system. A: Effects of MG132, pilsicainide and lidocaine on ubiquitinated p53-FLAG protein. Anti-FLAG immunoprecipitates (IP) were subjected to immunoblotting (IB) with the indicated antibodies in the presence of MG132 (50 μM), pilsicainide (20 μM) and lidocaine (1 mM). B: Effects of MG132 and LAs on p53-FLAG in COS7 cells expressing the dominant negative form of Mdm2. Transfected cells were cultured for 12 h in the presence of MG132 (50 μM), pilsicainide (20 μM), lidocaine (1 mM) or vehicle. Cells were transfected with p53-FLAG, GFP and Mdm2 (C438A) constructs. Mdm2, p53-FLAG, and GFP expression were confirmed by Western blot analysis and each protein level (upper panel) and ubiquitination (lower panel) of p53-FLAG was analyzed using the depicted antibodies.
